# Bariatric Surgery and Risk of End-Stage Liver Disease in a Four-Country Cohort Study

**DOI:** 10.1007/s11695-025-07857-x

**Published:** 2025-04-12

**Authors:** Dag Holmberg, Giola Santoni, My von Euler-Chelpin, Joonas H. Kauppila, Eivind Ness-Jensen, Jesper Lagergren

**Affiliations:** 1 Department of Molecular Medicine and Surgery, Karolinska Institute, and Karolinska University Hospital, Stockholm, Sweden; 2 Department of Public Health, University of Copenhagen, Copenhagen, Denmark; 3 Department of Surgery, Oulu University Hospital and University of Oulu, Oulu, Finland; 4 Department of Public Health and Nursing, NTNU, Norwegian University of Science and Technology, Trondheim/Levanger, Norway; 5 Medical Department, Levanger Hospital, Nord-Trøndelag Hospital Trust, Levanger, Norway; 6 School of Cancer and Pharmaceutical Sciences, King’s College London, London, UK

**Keywords:** Obesity, MASLD, Weight loss, Metabolic syndrome, Cirrhosis

## Abstract

**Background:**

Metabolic dysfunction-associated steatotic liver disease can progress to end-stage liver disease. Whether bariatric surgery influences this risk is uncertain.

**Methods:**

This population-based cohort study included all patients with an obesity diagnosis between 1989 and 2020 according to the nationwide patient registries in Denmark, Finland, Norway, and Sweden. Bariatric surgery was compared with non-operative care for incidence and mortality in end-stage liver disease. Patients with a history of alcohol overconsumption or liver disease at baseline were excluded. Multivariable Cox regression provided hazard ratios (HR) with 95% confidence intervals (CI), adjusted for sex, calendar year, diabetes, Charlson comorbidity index, and country.

**Results:**

Among 654,409 participants with obesity, 86,356 (12.6%) underwent bariatric surgery. During a follow-up period of up to 31 years (median 7.3 years), bariatric surgery was followed by increased incidence (HR 1.23, 95% CI 1.11–1.37) and mortality (HR 1.93, 95% CI 1.56–2.38) in end-stage liver disease compared to non-operative care. The incidence decreased between 1 and 5 years after bariatric surgery but thereafter increased. Censoring patients who developed specific liver diseases (not steatotic liver disease) or alcohol overconsumption during follow-up canceled the association (HR 0.92, 95% CI 0.70–1.06). The mortality in end-stage liver disease was similar after bariatric surgery compared to non-operative care within 1–5 years of follow-up, but thereafter more than twofold increased. Analyses restricted to gastric bypass showed similar associations regarding both incidence (HR 1.26, 95% CI 1.11–1.43) and mortality (HR 2.20, 95% CI 1.72–2.82) of end-stage liver disease.

**Conclusion:**

Bariatric surgery might be followed by an increased risk of end-stage liver disease.

**Supplementary Information:**

The online version contains supplementary material available at 10.1007/s11695-025-07857-x.

## Introduction

Metabolic dysfunction-associated steatotic liver disease (MASLD) is characterized by the deposition of fat in hepatocytes (steatosis) in the absence of alcohol overconsumption and is closely associated with obesity. MASLD has a global prevalence of 25% in adults overall and 80–90% among individuals with morbid obesity (body mass index ≥ 35), thus making it the most common type of liver disease [1–3]. MASLD and its subtype steatohepatitis are increasingly recognized as an important contributor to end-stage liver-disease [4, 5]. There is no specific treatment for MASLD, but patients with MASLD and obesity are recommended to lose weight to prevent progression to more severe liver disease [1].

Bariatric surgery is an intervention that results in both substantial and sustainable weight loss in individuals with morbid obesity [6]. Patients who undergo bariatric surgery often lose 25–30% of their body weight, which is far superior to non-operative weight-reducing methods both in the short and long term [7]. A systematic review and meta-analysis found that bariatric surgery results in histologic improvements in MASLD [8]. On the other hand, substance addiction may be increased after bariatric surgery which might lead to serious liver disease [9, 10]. Thus, bariatric surgery may prevent or promote the development of end-stage liver disease in patients with MASLD. Only two studies have examined how bariatric surgery influences the long-term risk of developing end-stage liver disease, one showing a decreased risk and the other an unchanged risk [11, 12].

This study is aimed at clarifying if and how bariatric surgery changes the risk of end-stage liver disease by using a large cohort of patients with obesity diagnosis with over three decades of follow-up.

## Materials and Methods

### Design

This population-based cohort study included all adults with a recorded obesity diagnosis in any of the four Nordic countries Denmark, Finland, Norway, or Sweden (alphabetic order). The overall study period was from January 1, 1989, until December 31, 2020, but differed between the participating countries (July 1, 1996, until December 31, 2019, in Denmark; January 1, 1989, until December 31, 2019, in Finland; January 1, 2007, until December 31, 2020, in Norway; and January 1, 1989, until December 31, 2020, in Sweden). Denmark started follow-up with the introduction of operation codes for bariatric surgery, which became available in 1996. Finland and Sweden started follow-up with the introduction of ICD- 9. Finally, the follow-up in Norway started with the introduction of the patient registry in 2007. The study was approved by the appropriate ethical review boards, data inspectorates, and governmental agencies in the participating countries [13].

### Obesity Cohort

The study cohort was an updated version of the Nordic Obesity Surgery Cohort (NordOSCo), which has been described in detail elsewhere [13, 14]. NordOSCo includes all patients with an obesity diagnosis or bariatric surgery in any of the national patient registries of Denmark, Finland, Norway, or Sweden. The patient registries in all participating countries record individual-level data on all hospital-based healthcare, including patient information, diagnoses, and procedures, and have a similar structure and quality, thus allowing for harmonization of data in large databases such as NordOSCo. In brief, the cohort of the present study consisted of all adult (≥ 18 years) individuals with an obesity diagnosis (ICD-codes are provided in Supplementary Table 1) according to any of the nationwide patient registries in the participating countries. The obesity diagnosis was made by at least one physician and registered in one of the participating countries’ patient registries. Data on all diagnoses and surgical procedures, cancer, and mortality came from nationwide complete, well-maintained, and well-validated registries [13]. Excluded were patients with a history of excessive alcohol consumption, any liver disease other than steatotic liver disease, or liver surgery (Supplementary Table 1).

### Exposure

The main study exposure was primary bariatric surgery, including the procedures gastric bypass, gastric banding, sleeve gastrectomy, and duodenal switch (Supplementary Table 1). The secondary exposure was gastric bypass alone, which was the dominating bariatric procedure in the Nordic countries during the study period. Bariatric surgery was compared with non-operative care for morbid obesity and related comorbidities, including advice and medical treatment. Data on bariatric surgery came from the national patient registries, which have been validated for high completeness and accuracy [15–17]. The patient registry in Sweden has specifically been validated for bariatric procedures by comparing registry data with medical records and operation charts in 938 patients, showing 97% concordance [18].

### Outcomes

The main outcome was incidence of end-stage liver disease. The secondary outcome was mortality in end-stage liver disease. End-stage liver disease included liver cirrhosis, esophageal or gastric varices, liver encephalopathy, hepatorenal syndrome, portal hypertension, ascites due to liver disease, liver failure, and liver transplantation. These diagnoses were identified from the corresponding diagnosis codes in the national patient registries (Supplementary Table 1). Mortality in end-stage liver disease was defined as death with one of the above-listed diagnoses as the main cause of death in the national cause of death registries, which have > 96% completeness for the cause of death [13].

### Statistical Analysis

Follow-up started 1 year after the date of obesity diagnosis (cohort entry). This 1-year time latency allowed for a lead time between the exposure attainment (bariatric surgery) and occurrence of outcomes. Thus, person-time and cases in both exposed and unexposed cohorts that occurred during this first year of follow-up were excluded from the analyses. Patients with obesity diagnosis who underwent bariatric surgery after the 1-year latency period were censored from the non-operated group and instead contributed person-time in the operated group from the date of surgery onwards, meaning that an individual could contribute time at risk in both groups. Follow-up ended at the date of end-stage liver disease diagnosis (for the main outcome only), death, or end of the study period, whichever occurred first. Cox proportional hazards regression was used to calculate hazard ratios (HR) with 95% confidence intervals (CI) for both outcomes. The time metric for the analysis was age at the date of obesity diagnosis or bariatric surgery [19, 20]. Hence, all analyses were adjusted for age and accounted for left truncation of the data due to unknown onset of obesity. A multivariable model adjusted for another five potential confounders: sex (female or male), calendar year (continuous), diabetes (yes or no), Charlson comorbidity index (excluding diabetes) (0, 1, or ≥ 2), and country (Denmark, Finland, Norway, or Sweden). Diabetes and Charlson comorbidity score were defined by the corresponding diagnosis codes in the national patient registries (Supplementary Table 1). The analyses were stratified by duration of follow-up (1–5, 5–10, 10–15, and ≥ 15 years), age (below and above the median), sex (female and male), and diabetes (yes or no).

To evaluate if any identified association was explained by differential development of liver diseases other than steatotic liver disease among the operated and non-operated patients, we conducted an analysis where patients were censored upon developing excessive alcohol consumption or liver diseases other than MASLD or non-alcoholic steatohepatitis during follow-up (Supplementary Table 2).

The proportional hazard assumption was not met for all analyses, but we stratified the HR by follow-up time which relaxed the assumption of proportional hazards. All analyses were determined in a detailed study protocol completed prior to data analysis. The data management and statistical analyses were conducted by a senior biostatistician (GS) using the statistical software STATA/MP 15.1.

## Results

### Patients

**Fig. 1:**
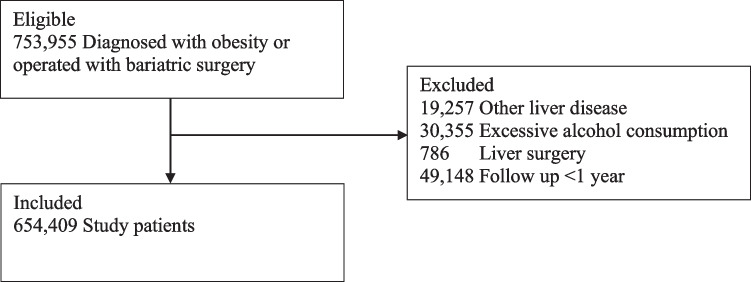
Selection of study patients

The study included 654,409 patients with an obesity diagnosis (Fig. 1). Of these, 86,356 (12.6%) underwent bariatric surgery during the study period and predominantly gastric bypass (*n* = 67,668; 78.4%). For the whole cohort, the mean age was 46.2 years (standard deviation, 16.1 years) and 72% were female. Patients who underwent bariatric surgery were slightly younger, more often women, and had a lower Charlson comorbidity index than non-operated patients (Table 1). During follow-up (maximum 31 years and median 7.3 years (interquartile range, 3.9–11.3)), 3459 (0.5%) participants developed end-stage liver disease and 776 (0.1%) died due to end-stage liver disease.

**Table 1:** Characteristics of 654,409 patients with obesity diagnosis, without and with bariatric surgery

Characteristic	No bariatric surgery	Bariatric surgery	
		Any type*	Gastric bypass
	Number (%)	Number (%)	Number (%)
**All patients**	600,927 (87.4)	86,356 (12.6)	67,668 (9.9)
**Age,** mean (SD)	46.8 (16.7)	41.8 (10.6)	41.9 (10.5)
18–30 years	128,138 (21.3)	14,236 (16.5)	10,856 (16.0)
31–45 years	175,718 (29.2)	40,241 (46.6)	31,728 (46.9)
46–60 years	152,161 (25.3)	28,880 (33.4)	22,811 (33.7)
> 61 years	144,910 (24.1)	2999 (3.5)	2273 (3.4)
**Sex**			
Female	430,589 (71.6)	65,217 (75.5)	51,022 (75.4)
Male	170,338 (28.4)	21,139 (24.5)	16,646 (24.6)
**Calendar year**			
1989–2005	114,981 (19.1)	10,165 (11.8)	2762 (4.1)
2006–2010	175,780 (29.3)	27,205 (31.5)	24,951 (36.9)
2011–2015	198,283 (33.0)	34,272 (39.7)	30,952 (45.7)
2016–2019	111,883 (18.6)	14,714 (17.0)	9003 (13.3)
**Diabetes**			
Yes	98,471 (16.4)	13,417 (15.5)	11,295 (16.7)
No	502,456 (83.6)	72,939 (84.5)	56,373 (83.3)
**Charlson comorbidity index**			
0	372,029 (61.9)	63,829 (73.9)	49,545 (73.2)
1	161,052 (26.8)	18,399 (21.3)	14,838 (21.9)
≥ 2	67,846 (11.3)	4128 (4.8)	3285 (4.9)
**Country**			
Denmark	259,889 (43.2)	16,842 (19.5)	14,513 (21.5)
Finland	101,430 (16.9)	7750 (9.0)	5802 (8.6)
Norway	22,322 (3.7)	3789 (4.4)	3151 (4.7)
Sweden	217,286 (36.2)	57,975 (67.1)	44,202 (65.3)
**Follow-up years,** mean (SD)	8.2 (5.3)	8.6 (5.8)	7.8 (3.8)
**End-stage liver disease**	3016 (0.5)	443 (0.5)	294 (0.4)
Liver cirrhosis	2981 (0.5)	440 (0.5)	291 (0.4)
Liver transplantation	76 (0.0)	12 (0.0)	6 (0.0)
Median time to outcome (IQR)	6.3 (3.6–10.3)	7.6 (5.2–12.2)	6.3 (4.3–8.5)
**Deaths**			
Total	70,871 (11.8)	3111 (3.6)	1714 (2.5)
From liver disease	659 (0.1)	117 (0.1)	79 (0.1)

*IQR*, interquartile range; *SD*, standard deviation

*Gastric bypass, gastric banding, sleeve gastrectomy, or duodenal switch

### Incidence of End-Stage Liver Disease

**Fig. 2:**
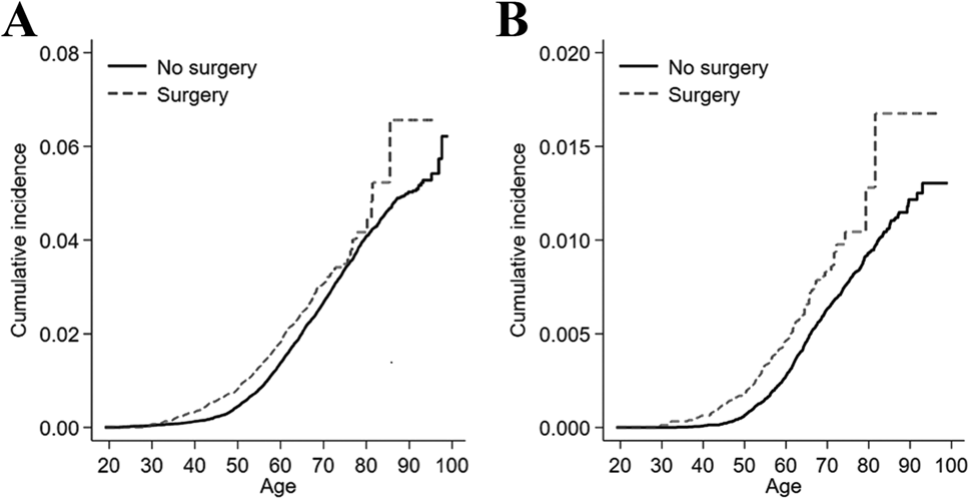
**A** Cumulative incidence of end-stage liver disease as a function of attained age in bariatric surgery versus non-operative care for morbid obesity. **B** Cumulative incidence of mortality in end-stage liver disease as a function of attained age in bariatric surgery versus non-operative care for morbid obesity

The cumulative incidence of end-stage liver disease among operated and non-operated patients with obesity diagnosis is presented in Fig. 2A. The incidence rate per 100,000 person-years of end-stage liver disease was 67.6 (95% CI 61.6–72.2) in operated patients and 70.2 (95% CI 67.7–72.7) in the non-operated patients. Bariatric surgery was followed by an overall 23% increased HR of end-stage liver disease compared to non-operative care (adjusted HR 1.23, 95% CI 1.11–1.37) (Table 2). The corresponding analysis of gastric bypass separately showed similar results (HR 1.26, 95% CI 1.11–1.43), and the point estimates after gastric bypass were consistently higher compared to after other bariatric procedures (Table 2).

**Table 2:** Hazard ratios (HR) with 95% confidence intervals (CI) of incidence of end-stage liver disease after bariatric surgery versus non-operative care for morbid obesity

	End-stage liver disease			
	Person-years	Cases (Number)	Crude HR (95% CI)	Adjusted HR (95% CI)*
**Bariatric surgery**				
** No**	4,297,606	3016	1.00 (Reference)	1.00 (Reference)
Yes**	654,970	443	1.19 (1.08–1.32)	1.23 (1.11–1.37)
Gastric bypass**	459,020	294	1.21 (1.07–1.37)	1.26 (1.11–1.43)
Other***	195,959	149	1.17 (0.99–1.38)	1.19 (0.99–1.42)
** No surgery**	4,297,606	3016	1.00 (Reference)	1.00 (Reference)
**Years after surgery**				
1–5	295,896	106	0.78 (0.64–0.95)	0.79 (0.65–0.97)
5–10	224,031	185	1.44 (1.23–1.67)	1.46 (1.12–1.70)
10–15	73,756	69	1.35 (1.06–1.72)	1.43 (1.12–1.82)
> 15	61,288	83	1.46 (1.17–1.81)	1.66 (1.31–2.10)
**Women**				
No surgery	3,089,516	1223	1.00 (Reference)	1.00 (Reference)
Surgery	500,831	297	1.66 (1.46–1.89)	1.52 (1.33–1.74)
**Men**				
No surgery	1,208,090	1793	1.00 (Reference)	1.00 (Reference)
Surgery	154,138	146	0.93 (0.78–1.10)	0.91 (0.77–1.08)
**Age < 45 years**				
No surgery	2,146,753	470	1.00 (Reference)	1.00 (Reference)
Surgery	417,987	227	1.89 (1.61–2.22)	1.79 (1.52–2.11)
**Age ≥ 45 years**				
No surgery	2,150,852	2546	1.00 (Reference)	1.00 (Reference)
Surgery	236,983	216	0.88 (0.76–1.02)	0.95 (0.82–1.10)
**No diabetes**				
No surgery	3,626,230	1821	1.00 (Reference)	1.00 (Reference)
Surgery**	570,118	350	1.41 (1.26–1.59)	1.42 (1.26–1.61)
**Diabetes**				
No surgery	671,377	1195	1.00 (Reference)	1.00 (Reference)
Surgery	84,852	93	0.78 (0.63–0.97)	0.84 (0.68–1.05)

*Adjusted for sex, calendar year, diabetes, Charlson comorbidity index, and country

**Not proportional HR in the adjusted model

***Gastric bypass, gastric banding, sleeve gastrectomy, or duodenal switch

When stratified by follow-up periods, the HR was initially decreased 1–5 years after bariatric surgery (adjusted HR 0.79, 95% CI 0.65–0.97) but was increased in all later follow-up periods and was 1.66 (95% CI 1.31–2.10) after > 15 years of follow-up (Table 2). Other stratified analyses revealed that women (HR 1.52, 95% CI 1.33–1.74), participants aged < 45 years (HR 1.79, 95% CI 1.52–2.11), and non-diabetics (HR 1.42, 95% CI 1.26–1.61) who underwent bariatric surgery were at statistically significantly increased risks of end-stage liver disease compared to non-operated participants. No subgroup benefitted from bariatric surgery with regard to the risk of developing end-stage liver disease (Table 2). The effects of the interaction terms are presented in Supplementary Tables 3 and 4.

In the analysis censoring for liver diseases (others than MASLD and non-alcoholic steatohepatitis) diagnosed during the follow-up, the association between bariatric surgery and end-stage liver disease did not remain (HR 0.92, 95% CI 0.79–1.06).

### Mortality from End-Stage Liver Disease

The cumulative mortality from end-stage liver disease among operated and non-operated patients is presented in Fig. 2B. Bariatric surgery was associated with a 93% increased HR of mortality from end-stage liver disease compared to non-operative care (adjusted HR 1.93, 95% CI 1.56–2.38) (Table 3). The risk was increased also in analyses restricted only to patients who underwent gastric bypass (adjusted HR 2.20, 95% CI 1.72–2.82).

**Table 3:** Hazard ratios (HR) with 95% confidence intervals (CI) of mortality in end-stage liver disease after bariatric surgery versus non-operative care for morbid obesity

	Liver disease-specific mortality			
	Person-years	Cases (number)	Crude HR (95% CI)	Adjusted HR (95% CI)*
**Bariatric surgery**				
** No**	4,307,395	705	1.00 (Reference)	1.00 (Reference)
Yes**	656,650	121	1.43 (1.17–1.74)	1.93 (1.56–2.38)
Gastric bypass**	460,062	83	1.51 (1.19–1.91)	2.20 (1.72–2.82)
Other***	196,588	38	1.28 (0.92–1.78)	1.51 (1.06–2.14)
** No surgery**	4,307,395	705	1.00 (Reference)	1.00 (Reference)
**Years after surgery**				
1–5	296,340	22	0.74 (0.48–1.13)	1.06 (0.69–1.64)
5–10	224,623	59	1.99 (1.52–2.61)	2.64 (2.00–3.49)
10–15	74,036	19	1.59 (1.00–2.51)	2.00 (1.25–3.19)
> 15	61,651	21	1.54 (1.00–2.38)	2.04 (1.27–3.25)
**Women**				
No surgery	3,093,974	246	1.00 (Reference)	1.00 (Reference)
Surgery	501,939	77	2.17 (1.67–2.81)	2.60 (1.98–3.40)
**Men**				
No surgery	1,213,422	459	1.00 (Reference)	1.00 (Reference)
Surgery	154,711	44	1.14 (0.83–1.55)	1.38 (1.00–1.89)
**Age < 45 years**				
No surgery	2,148,950	90	1.00 (Reference)	1.00 (Reference)
Surgery	418,761	55	2.21 (1.58–3.09)	2.90 (2.05–4.09)
**Age ≥ 45 years**				
No surgery	2,158,446	615	1.00 (Reference)	1.00 (Reference)
Surgery	237,889	66	1.12 (0.86–1.45)	1.56 (1.19–2.04)
**No diabetes**				
No surgery	3,632,579	442	1.00 (Reference)	1.00 (Reference)
Surgery**	571,334	92	1.58 (1.25–1.99)	2.13 (1.68–2.71)
**Diabetes**				
No surgery	674,816	263	1.00 (Reference)	1.00 (Reference)
Surgery	85,316	29	1.12 (0.76–1.65)	1.53 (1.03–2.26)

*Adjusted for sex, calendar year, diabetes, Charlson comorbidity index, and country

**Not proportional HR in the adjusted model

***Gastric banding, sleeve gastrectomy, or duodenal switch

Stratification by follow-up periods indicated that the risk was similar to non-operated patients 1–5 years after surgery (HR 1.06, 95% CI 0.69–1.64) but was at least twofold increased after bariatric surgery within each of the longer follow-up periods, and the HR was 2.04 (95% CI 1.27–3.25) in the > 15 years of follow-up category (Table 3). Increased mortality from end-stage liver disease after bariatric surgery was found in both sexes, all age groups, and patients with and without diabetes (Table 3).

## Conclusion

This study indicates that bariatric surgery, predominantly gastric bypass, is associated with an increased risk of end-stage liver disease 5 years after surgery.

Methodological strengths of the study include the large cohort size, the population-based design with high participation rates, and the long and complete follow-up. The data came from well-maintained registries which have been extensively validated for high completeness and quality. The robustness of the findings was confirmed in analyses adjusted by country (and other variables) showing similar results despite some differences in the management of obesity, bariatric surgery, and liver disease across the countries. Thus, the study mirrors real-world data from the Nordic countries and should be free of much bias from selection or misclassification. The findings may therefore be generalizable to countries with similar demographics and healthcare. Among weaknesses of the observational design used is possible confounding. However, we homogenized the operated and non-operated groups by excluding all liver diseases at baseline and adjusted the results for several key variables. Direct data on some other potentially relevant variables were not available, i.e., baseline fibrosis status, impaired fasting glucose, alcohol consumption, and body mass index, but these were partly controlled for by adjusting for diabetes and Charlson comorbidity index and by excluding patients with alcohol-related diagnoses at baseline. Patients who are selected for surgery may undergo more rigorous screening for alcohol overconsumption, particularly in more recent calendar years because the awareness of postoperative alcohol overconsumption has been more widely known among surgeons. Thus, we cannot exclude residual confounding from these variables. The non-operated comparison group represents only a proportion of all individuals with morbid obesity in the Nordic countries, and those included might be the most severely obese and those with the most serious comorbidities, which was reflected by the higher Charlson comorbidity index score. However, such selection bias would not explain the higher risk of end-stage liver disease in the operated compared to the non-operated participants found in the study but rather lead to an underestimation of this association. Likewise, we cannot exclude that the non-operated comparison group included individuals with a lower body mass index than patients in the surgery group.

Research has indicated that patients operated with bariatric surgery, and particularly those who undergo gastric bypass, are prone to developing alcohol addiction postoperatively [9, 21]. Unphysiologically rapid transit and absorption of alcohol to the bloodstream increases the potency of ingested alcohol and contributes to the increased risk of postoperative alcohol addiction and associated liver injury [22]. The present study indicated that excessive alcohol consumption and liver diseases other than MASLD or steatohepatitis could be mediators of the increased risk of end-stage liver disease after bariatric surgery. This mechanism is also indirectly supported by the more pronounced risk of end-stage liver disease in women and younger individuals, which are more susceptible to alcohol-induced liver toxicity and more likely to require liver transplantation due to alcoholic liver cirrhosis after bariatric surgery [23]. Additionally, the association between bariatric surgery and end-stage liver disease was more pronounced in patients without diabetes, which is inversely related to alcohol overconsumption [24]. Nevertheless, these results build on indirect data from censoring of patients with alcohol overconsumption rather than direct measurements of postoperative alcohol consumption, meaning that they should be interpreted with caution. We also found that the risk of end-stage liver disease varied over different periods of follow-up. In the first 1–5 years of follow-up, bariatric surgery seemed protective, but as time wore on, the risk associated with bariatric surgery increased. The inverse association occurred during the same time period as the weight loss and catabolic state induced by bariatric surgery, which could explain a reduced incidence of end-stage liver disease in this time period.

Only two previous studies have examined how bariatric surgery influences the risk of liver disease. A US cohort study of 1158 patients with non-alcoholic steatohepatitis found a lower 10-year incidence of end-stage liver disease in those 650 patients who underwent bariatric surgery (2.3%, 95% CI 0.0–4.6%) compared to 508 non-operated patients (9.6%, 95% CI 6.1–12.9%) [11], while a Swedish cohort study found that 1942 patients who underwent bariatric surgery had a similar long-term risk of severe liver disease compared to 1980 non-operated patients with obesity (HR 0.96, 95% CI 0.66–1.41) [12]. The present larger study instead found an increased long-term risk of end-stage liver disease following bariatric surgery compared to non-operated patients with obesity. These differences warrant a discussion. The US study included patients with histologically verified steatohepatitis, which represent only a minor subgroup of MASLD patients [11]. Because patients with steatohepatitis are at a substantially increased risk of end-stage liver disease, they are usually followed up annually with physical examinations, blood tests, and are dissuaded from using alcohol, which might induce further liver damage [25, 26]. It is therefore likely that alcohol habits were markedly lower in patients with steatohepatitis, especially in probably more well-motivated participants who underwent bariatric surgery compared to those who did not. In the Swedish study, patients were monitored closely for up to 20 years, including assessment of postoperative alcohol consumption. This is markedly different from routine clinical practice, mirrored in the present study, where operated patients are followed up only during the initial period of postoperative weight loss. Additionally, the Swedish study enrolled patients in the early years of bariatric surgery (1987–2001) when the selection of patients was different compared to the more modern setting, with less rigorous follow-up of alcohol overconsumption [12]. Additionally, the incidence rate of end-stage liver disease was higher in both operated and unoperated patients in the Swedish intervention study. A possible explanation for this is the longer median follow-up time in that study, which was the consequence of limiting the recruitment period to 1987–2001, as incidence rates tended to increase with longer follow-up. The increased risk of end-stage liver disease observed in the present study may thus better reflect a more current and unselected healthcare where patients are not closely monitored postoperatively and where the increased risk of alcohol overconsumption offsets the benefits of weight loss induced by bariatric surgery.

Assuming future research will confirm the results of this study, there are some clinical implications for this study. First, a decreased risk of end-stage liver disease would not be expected following bariatric surgery but rather the opposite. Second, it would be vital to dissuade bariatric surgery patients from using alcohol and to identify those who nevertheless overconsume alcohol, for example, using serum biomarkers of alcohol overconsumption. We excluded patients with alcohol-related disorders at baseline, but there remains a possibility that some patients still overconsumed alcohol at the time of surgery.

To conclude, this large multi-country and population-based cohort study with long and complete follow-up and adjustment for confounders found an increased incidence and mortality in end-stage liver disease after bariatric surgery compared to non-operative management of morbid obesity. This increase seems to be mediated by the postoperative development of other liver diseases than MASLD.

## Supplementary Information

Below is the link to the electronic supplementary material.

Supplementary file1 (DOCX 28 KB)

## Data Availability

Data are available from the registry holders and cannot be shared for legal reasons.
