# Supplementary material for: Bariatric Surgery and Risk of End-Stage Liver Disease in a Four-Country Cohort Study
Source: Obes Surg. 2025 Apr 12;35(5):1845–53. doi: 10.1007/s11695-025-07857-x (PMC12065758; doi:10.1007/s11695-025-07857-x)
Supplement: Supplementary file 1 — Supplementary file1 (DOCX 28 KB) [file 11695_2025_7857_MOESM1_ESM.docx]

**SUPPLEMENTARY MATERIALS: Bariatric surgery and risk of end-stage liver disease in a four-country cohort study**

| **Supplementary Table 1. Diagnosis codes using International Classification of Diseases (ICD) and procedural codes using NOMESCO for defining the study cohort.** | | | | |
| --- | --- | --- | --- | --- |
|  | **Inclusion criteria** | | | |
|  | **ICD-10** | | **ICD-9** | **ICD-8** |
| **Obesity** | E66 | | 278A-B, 2780-1 | 277 |
|  |  | | | |
|  | **Exclusion criteria** | | | |
|  | **ICD-10** | | **ICD-9** | |
| **Viral hepatitis** | B15-B19 | | 070 | |
| **Other** | I85, I86.4, I98.2, K70-K77 (except K76.0 (NAFLD)), E830B, E831, E880, C22-C25, C787, R16.2, Z94.4 | | 570-573 (except 571W (NAFLD)), 275B, 275A, 277G, 155-157, 197H, 456A-C | |
| **Excessive alcohol consumption** | F10, G31.2, G62.1, G72.1, I42.6, K29.2, K70, K86.0, Z71.4, X45, Y15, T51.0, T51.1, T51.9, E51, E52 | | 291, 303, V79B, 305A, 357A, 359E, 425F, 535D, 571, 572W, 577B, V65E, E860, E862, E980, 980A, 980B, 980X, 265 | |
|  | **NOMESCO (1997-)** | | **Old classifications (-1996)** | |
|  |  | | **Sweden** | **Finland** |
| **Liver surgery** | JJ | | 5100-5282 | 6601-6604  6611-6615  6619 |
|  |  | | | |
|  | **Exposure** | | | |
|  | **NOMESCO (1997-)** | | **Old classifications (-1996)** | |
|  |  | | **Sweden** | **Finland** |
| **Vertical-Banded Gastroplasty** |  | | 4751 |  |
| **Gastric bypass** | JDF10-11, JDF50-51 | | 4752 |  |
| **Gastric banding** | JDF20-21 | | 4753 |  |
| **Sleeve gastrectomy** | JDF96 (after 2000), JDF97 (after 2000), JDF40-41, JDF00-01 (after 2000) | | - | - |
| **Other** | JDF00-01 | | 4759 | 6548, 6559 |
| **Duodenal shunt with biliopancreatic diversion** | JFD03-04 | | 4750 |  |
|  |  | | | |
|  | **Outcome** | | | |
| **Unspecified liver cirrhosis (not alcohol-related)** | | K746 | 571F | |
| **Ascites** | | R18 | 789F | |
| **Esophageal varices** | | I85, I98.2 | 456A-C | |
| **Gastric varices** | | I86.4 | - | |
| **Liver encephalopathy** | | - | 572C | |
| **Hepatorenal syndrome** | | K767 | 572E | |
| **Portal hypertension** | | K766 | 572D | |
| **Liver failure** | | K729 | 570 | |
| **Previous liver transplantation** | | Z94.4 | - | |
|  | | **NOMESCO (1997-)** | **Old classifications (-1996)** | |
|  | |  | **Sweden** | **Finland** |
| **Liver transplantation** | | JJC00, JJC10, JJC20, JJC30, JJC40 | 5200, 5202 | 6615 |
|  | |  | | |
|  | | **Comorbidities** | | |
|  | | **ICD-10** | **ICD-9** | **ICD-8** |
| **Myocardial Infarction** | | I21*-I23*, I252* | 410*, 412* | 410*, 412* |
| **Congestive Heart Failure** | | I11*, I13*, I255*, I42*-I43*, I50*, I517* | 402*, 425*, 428*, 429D*^¥^ | 427*, 428* |
| **Peripheral Vascular Disease** | | I70*-I73*, I770*-I771*, K551*, K558*- K559*, R02*, Z958*-Z959* | 440*-447*, V43E*^¥^, 785E*^¥^ | 440*- 445* |
| **Cerebrovascular disease** | | G45*-G46*, I60*-I69* | 430*-438*, 362D*^¥^ | 430*- 438* |
| **Dementia** | | A810*, F00*-F03*, F051*, G30*-G31* | 290*, 294B*^¥^ | 290* |
| **Chronic pulmonary disease** | | I26*-I27*, J40*-J47, J60*-J67*, J684*, J701*, J703* | 490*-496*, 500*-505*, 416*,  506E*^¥^ | 490*- 493*, 515*- 518* |
| **Rheumatic disease** | | M05*-M06*, M09*, M120*, M315*, M32*-M36* | 710*-714*, 725* | 710*-712*, 734* |
| **Liver disease** | | B18*, I85*, I864, I982*, K70*-K71*, K721*, K729*, K76*, R162*, Z944* | 070*, 571* - 573*  456A*^¥^-456C*^¥^ | 070*, 4560*, 571*, 573* |
| **Diabetes mellitus** | | E10*-E14* | 250* | 250* |
| **Hemiplegia** | | G114*, G81*-G83* | 342*-344* | 344* |
| **Renal disease** | | I12*-I13*, N01*, N03*, N05*, N07*-N08*, N171*-N172*, N18*, N19*, N25*, Z49*, Z940*, Z992* | 403*- 404*, 580-588*, V42A*^¥^, V45B*^¥^ | 403* - 404*, 580* - 583*, 792* |
| **Any malignancy** | | C00*–C26*, C30*–C34*, C37*–C41*, C43*, C45*-C58*, C60–C76*, C80*-C85*, C88*, C90*-C97* | 140*-172*, 174*-195*, 200*-208* | 140*- 172*, 174* - 195*, 200*-207* |
| **Metastatic tumors** | | C77*-C79* | 196*-199* | 196* - 199* |
| **AIDS** | | B20*–B24* | 279K*^¥^ | N/A |
| * All positions that follow are valid without need of further specification.  ¥ Finland uses a variation of this code. | | | | |

| **Supplementary Table 2. Codes used for censoring liver diseases in sensitivity analysis.** | | | | |
| --- | --- | --- | --- | --- |
|  | **ICD-10** | **ICD-9** | | **ICD-8** |
| **Viral hepatitis** | B15-B19 | 070 | | 070 |
| **Liver diseases** | K70-K77 (except K74.6, K75.8, K76.0, K76.6, K72.9,) E830B, E831, E880, C22-C25, C787 | 571-573 (except 571W, 571F, 572C, 572D, 572E), 275B, 275A, 277G, 155-157, 197H | | 571-573 (except 57100, 5719), 2732, 2733, 155-157, 1977, 1978 |
| **Excessive alcohol consumption** | F10, G31.2, G62.1, G72.1, I42.6, K29.2, K70, K86.0, Z71.4, X45, Y15, T51.0, T51.1, T51.9, E51, E52 | 291, 303, V79B, 305A, 357A, 359E, 425F, 535D, 571, 572W, 577B, V65E, E860, E862, E980, 980A, 980B, 980X, 265 | | 291, 57100, 57101, 57710, 57719, 303, 26100, 26109, 26200 |
|  | **NOMESCO** | **Old classifications** | | |
|  |  | **SWE** | **FIN** | |
| **Liver surgery** | JJ (except JJC00, JJC10, JJC20, JJC30, JJC40) | 5100-5282 (except 5200, 5202) | 6601-6604  6611-6614  6619 | |

| **Supplementary Table 3. Hazard ratios (HR) with 95% confidence intervals (CI) of incidence of end-stage liver disease after bariatric surgery versus non-operative care for morbid obesity; results from the interaction analysis.** | | | | | |
| --- | --- | --- | --- | --- | --- |
|  | **End-stage liver disease** | | | |  |
|  | **Person-years** | **Cases (Number)** | **Crude HR**  **(95% CI)** | **Adjusted HR**  **(95% CI)*** |  |
| **Women** |  |  |  |  |  |
| No surgery | 3,089,516 | 1,223 | 1.00 (Reference) | 1.00 (Reference) |  |
| Surgery | 500,831 | 297 | 1.66 (1.46-1.89) | 1.52 (1.33-1.74) |  |
| **Men** |  |  |  |  |  |
| No surgery | 1,208,090 | 1,793 | 1.00 (Reference) | 1.00 (Reference) |  |
| Surgery | 154,138 | 146 | 0.93 (0.78-1.10) | 0.91 (0.77-1.08) |  |
| Interaction term** |  |  | 1.78 (1.44-2.20) | 1.67 (1.35-2.06) |  |
|  |  |  |  |  |  |
| **Age <45 years** |  |  |  |  |  |
| No surgery | 2,146,753 | 470 | 1.00 (Reference) | 1.00 (Reference) |  |
| Surgery | 417,987 | 227 | 1.89 (1.61-2.22) | 1.79 (1.52-2.11) |  |
| **Age ≥45 years** |  |  |  |  |  |
| No surgery | 2,150,852 | 2,546 | 1.00 (Reference) | 1.00 (Reference) |  |
| Surgery | 236,983 | 216 | 0.88 (0.76-1.02) | 0.95 (0.82-1.10) |  |
| Interaction term |  |  | 0.47 (0.38-0.58) | 0.53 (0.43-0.66) |  |
|  |  |  |  |  |  |
| **No diabetes** |  |  |  |  |  |
| No surgery | 3,626,230 | 1,821 | 1.00 (Reference) | 1.00 (Reference) |  |
| Surgery** | 570,118 | 350 | 1.41 (1.26-1.59) | 1.42 (1.26-1.61) |  |
| **Diabetes** |  |  |  |  |  |
| No surgery | 671,377 | 1,195 | 1.00 (Reference) | 1.00 (Reference) |  |
| Surgery | 84,852 | 93 | 0.78 (0.63-0.97) | 0.84 (0.68-1.05) |  |
| Interaction term** |  |  | 0.55 (0.43-0.70) | 0.59 (0.47-0.76) |  |
| * Adjusted for sex, calendar year, diabetes, Charlson comorbidity index, and country  ** Not proportional HR in the adjusted model. | | | | | |

| **Supplementary Table 4. Hazard ratios (HR) with 95% confidence intervals (CI) of mortality in end-stage liver disease after bariatric surgery versus non-operative care for morbid obesity; results from the interaction analysis.** | | | | |
| --- | --- | --- | --- | --- |
|  | **Liver disease-specific mortality** | | | |
|  | **Person-years** | **Cases (Number)** | **Crude HR (95% CI)** | **Adjusted HR (95% CI)*** |
| **Women** |  |  |  |  |
| No surgery | 3,093,974 | 246 | 1.00 (Reference) | 1.00 (Reference) |
| Surgery | 501,939 | 77 | 2.17 (1.67-2.81) | 2.60 (1.98-3.40) |
| **Men** |  |  |  |  |
| No surgery | 1,213,422 | 459 | 1.00 (Reference) | 1.00 (Reference) |
| Surgery | 154,711 | 44 | 1.14 (0.83-1.55) | 1.38 (1.00-1.89) |
| Interaction term** |  |  | 1.91 (1.28-2.81) | 1.89 (1.26-2.82) |
|  |  |  |  |  |
| **Age <45 years** |  |  |  |  |
| No surgery | 2,148,950 | 90 | 1.00 (Reference) | 1.00 (Reference) |
| Surgery | 418,761 | 55 | 2.21 (1.58-3.09) | 2.90 (2.05-4.09) |
| **Age ≥45 years** |  |  |  |  |
| No surgery | 2,158,446 | 615 | 1.00 (Reference) | 1.00 (Reference) |
| Surgery | 237,889 | 66 | 1.12 (0.86-1.45) | 1.56 (1.19-2.04) |
| Interaction term |  |  | 0.51 (0.33-0.78) | 0.54 (0.35-0.82) |
|  |  |  |  |  |
| **No diabetes** |  |  |  |  |
| No surgery | 3,632,579 | 442 | 1.00 (Reference) | 1.00 (Reference) |
| Surgery** | 571,334 | 92 | 1.58 (1.25-1.99) | 2.13 (1.68-2.71) |
| **Diabetes** |  |  |  |  |
| No surgery | 674,816 | 263 | 1.00 (Reference) | 1.00 (Reference) |
| Surgery | 85,316 | 29 | 1.12 (0.76-1.65) | 1.53 (1.03-2.26) |
| Interaction term** |  |  | 0.71 (0.46-1.10) | 0.72 (0.46-1.12) |
| * Adjusted for sex, calendar year, diabetes, Charlson comorbidity index, and country  ** Not proportional HR in the adjusted model. | | | | |
